# Socio-ecological influences on adolescent dietary typologies

**DOI:** 10.1177/02601060231186297

**Published:** 2023-07-10

**Authors:** Hannah C. Greatwood, Suzanne McGregor, Lauren C. Duckworth, Claire Griffiths

**Affiliations:** School of Sport, 4467 Leeds Beckett University, Leeds, LS6 3QT, UK

**Keywords:** Latent class analysis, dietary behaviour, food, adolescents, millennium cohort study

## Abstract

**Background:** Dietary behaviours of adolescence are concerning, and this may impact long-term well-being. **Aim:** This study examined the socio-ecological determinants of dietary behaviours in a national prospective cohort study of English adolescents. **Methods:** Latent class analysis was used to identify the typologies of eight dietary behaviours: fruit, vegetable, breakfast, sugar-sweetened beverages, artificial-sweetened beverages, fast-food, bread, and milk from 7402 adolescents aged 13–15 years (mean 13.8 ± 0.45 years) (50.3% female and 71.3% white ethnicity) participating in the U.K. Millennium Cohort Study (sixth survey). Multinomial logistic regression and path analysis predicted associations between personal characteristics, individual, influential others, social environment and physical environment determinants and three distinct diet typologies: (1) healthy, (2) less-healthy and (3) mixed, (reference category = mixed). **Results:** Within Path analysis, the magnitudes of coefficients were small to moderate suggesting a relatively weak relationship between the variables. Model 1 reported adolescents within the less-healthy compared to mixed typology had lower levels of physical activity (β = 0.074, 95% CI = −0.115, −0.033), and have siblings (β = 0.246, 95% CI = 0.105, 0.387). Model 2 reported adolescents within the healthy compared to mixed typology had lower screen time (β = 0.104, 95% CI = 0.067, 0.141), and lower social media usage (β = 0.035, 95% CI = 0.024, 0.046). **Conclusion:** This study highlights the importance of considering multiple dietary determinants. These findings are likely to be useful in supporting the development of multi-faceted interventions. They emphasise the need to move away from investigating silo behaviours on individual diet components and a step towards more systems thinking to improve adolescent eating behaviours.

## Introduction

Poor dietary behaviours that led to non-communicable diseases, including cardiovascular disease, diabetes and cancer, were responsible for 11 million (22%) global adult deaths in 2017 (Afshin et al., 2019). To prevent their onset, changes in dietary practices are recommended. This is important during adolescence, a developmental period defined by the high nutritional demands required to fuel changes in physical, cognitive, and social-emotional characteristics (US Department of Health and Human Services, 2012). Evidence suggests that adolescents continue to make less than optimum dietary choices (Public Health England, 2020), and given the long-term impact of food choices on adult health (Vos et al., 2017), improving adolescent dietary behaviours is a national public health priority.

Despite major investments in improving the dietary behaviours of English adolescents, data from the National Diet and Nutrition Survey, suggest current intervention effectiveness is limited. For example, for the period 2016 to 2019, free sugar and saturated fat intakes exceeded the Government recommendations of providing no more than 5% (Public Health England, 2015) and 10% (Public Health England, 2019) of total energy intake by 7.3% and 2.6%, respectively (Public Health England, 2020). Rutter et al. (2017) suggest that the evidence underpinning tackling public health behaviours, including diet, has been grounded in linear models of cause and effect. To have a positive outcome, they should instead consider complex systems that conceptualise health behaviours as outcomes of a multitude of interdependent elements within a connected whole (Rutter et al., 2017). If we are to be successful in identifying, implementing, and evaluating effective changes in adolescents’ diets, lessons can be learned from the extensive work undertaken in related disciplines for example obesity (Bagnall et al., 2019). Reshaping dietary behaviour research, policy, and practice to recognise the complexity of the systems involved in behaviours may potentially improve adolescent diets and subsequent health.

Sleddens et al. (2015) identified environmental (mainly social-cultural) and social-cognitive determinants as key determinants of youth dietary behaviours, however, noted a paradigm shift from a social-cognitive approach towards a social-ecological method in the assessment of behavioural influences. The socio-ecological model (SEM) recognises the complexity of multiple behaviours on an outcome (Stokols, 1996) and emphasises the interaction between, and interdependence of, factors within and across multiple levels of behaviour. The model recognises the complex interplay between different layers, including personal characteristics, the individual, influential others (e.g., parents or friends), social environment, and physical environment. This concept moves away from targeting individual behaviours and considers multiple elements across the many systems that influence dietary intake to tackle health disparities (Bagnall et al., 2019; Rutter et al., 2017).

While insightful analyses have previously considered the layers of the SEM in isolation, most of the work to date has failed to consider the interplay of these layers. Given the complexity of influences on adolescent dietary behaviours, and the importance of targeting health, it is imperative that we consider the contribution of multiple predictors. In addition to recognising that behaviour is influenced by multiple factors, it is also important to recognise that food and nutrients are not consumed independently, and the use of dietary patterns to describe dietary behaviours has become increasingly popular in nutritional epidemiology (Dai et al., 2020). The SEM will provide a framework to investigate multiple influences on a variety of adolescent dietary behaviours. Investigating if these layers predict dietary typologies will assist in the development of effective and sustainable multi-faceted interventions for the promotion of healthy eating, which aim to have a positive effect on long-term public health.

## Methods

### Study procedure and participants

Cross-sectional data were analysed from wave 6 (2014) of the Millennium Cohort Study (MCS) (University of London, 2007). The MCS, is an observational cohort study, tracking U.K. children born in 2000 (*n *= 19,519). Full details of the study are published elsewhere (Connelly and Platt, 2014). Ethical approval for the MCS6 surveys was obtained by the Centre for Longitudinal Studies (CLS) and from the National Research Ethics Service (NRES) Research Ethics Committee (REC) London – Central (REC ref: 13/LO/1786).

### Measures

#### Dietary behaviours

Eight dietary behaviours; fruit, vegetable, sugar-sweetened beverages (SSB), artificially sweetened beverages (ASB), fast-food (FF), breakfast, bread and milk (supplement 1) were assessed via questionnaires administered to adolescents by trained interviewers in their homes. English adolescents who had complete data for the eight dietary behaviours assessed were included in the analysis (*n* = 179, 2.36% excluded). The national sample size was *n* = 7402.

#### Socio-ecological variables

Interviews with both adolescent and their caregiver were used to assess socio-ecological variables, including (1) personal characteristics (2) individual characteristics (3) influential others (4) social environment and (5) perceived physical environment. Supplement 1 outlines the full details of the questions.

##### Personal Characteristics

Three personal characteristics were included in the analysis; (1) sex, (2) ethnicity, (71.3% of the sample identified as being white, therefore, to ensure the stability of estimates within each group, all other ethnic minorities were cumulated into a non-white group (Beydoun and Wang, 2011; Griffiths et al., 2010)), (3) BMI classification (height, measured using a Leicester stadiometer and weight, measured using Tanita BF-522 W scales).

##### Individual

Adolescents were interviewed and reported on: (1) post-school intention, (2) perceived weight status, (3) screen time and social media usage, (4) ownership of a computer, (5) physical activity levels, (6) time at night spent asleep, (7) smoking status, (8) perceived well-being, (9) perceived health, (10) self-esteem (measured using questions taken from Rosenburg (1965)) and (11) cognitive ability (measured as a score out of 20, using a word activity from subsets used by the 1970 British Cohort Study and originally from standardised vocabulary tests devised by the Applied Psychology Unit at the University of Edinburgh in 1976 (Closs, 1976)).

##### Influential others

Adolescents were asked about the role of influential others in their lives and reported (1) the number of parents/carers in the household, which collapsed to one or two or more, and encompassed adoptive, foster, step, natural, and grandparents, (2) parental control, (3) the number of siblings in the house, (4) parental health, (5) frequency of consuming meals as a family, (6) time spent with close friends, and (7) hours spent on social media.

##### Social environment

In the caregiver interview, parents were asked four questions about their social environment: (1) parental education, (2) parental cognitive ability (measured using a word activity), (3) household income, and (4) home postcode, which was allocated a decile of deprivation using the Index of Multiple Deprivation (IMD, 2004).

##### Perceived physical environment

Data about the physical environment were not available. Both adolescents and their parents were asked about how safe they felt their neighbourhood was to be active during the day. Only 13 participants responded that they did not feel the area they lived in was ‘not very safe at all’, these results were combined with those participants that reported the area was ‘not very safe’.

##### Statistical Analysis

Analyses were performed using the statistical software programmes SPSS (*version 24; SPSS Inc, Chicago, IL*) and STATA MP (*version* 14.2). The alpha level adopted for statistical significance was *p* < 0.05. To detect collinearity, if a relationship existed between predictor variables, Pearson's correlation coefficient was used. Between variable correlations did not reach *r* ≥ 0.7, therefore, independent variables were assumed to not correlate among themselves (Vatcheva et al., 2016). Latent class analysis (LCA) derived mutually exclusive classes that maximised between-group variance and minimised within-group variance based on several model fit criteria. The expectation-maximisation algorithm was used for class derivation and assignment to identify participants who had similar combinations of dietary behaviours based on their responses to eight dietary questions. One to six classes/typologies were tested, and the ideal model was selected based on model fit statistics of the Bayesian Information Criterion (BIC), which includes sample sizes per class, usefulness, and substantive interpretation. The number of classes was selected using a combination of parsimony and interpretability.

Multinomial logistic regression was used to predict LCA groups with socio-ecological variables. All socio-ecological variables were entered into the regression model using a stepwise method to predict the dietary behaviour typologies. Stepwise methods can automatically select the variables that will influence the model, that is, at each step, the term whose addition causes the largest statistically significant change in the −2 log-likelihood is added to the model. The final model included the significant predictors of the outcome variable. Using the variables identified as significant, a path analysis was conducted to assess the estimates of the magnitude and the significance of the hypothesised connections between socio-ecological predictors and dietary typologies. A structural equation model builder was used to draw out the models using the observed variables and the proposed pathways. Maximum likelihood was used to assess *p* values, 95% CI and coefficient estimates.

## Results

Prior to the publication of the data by CLS two variables were imputed: (1) ethnicity, since it is a fixed attribute over time and (2) parental education qualification (Mostafa and Ploubidis, 2017). Cases were excluded for the analysis within this study if they did not have data for all dietary behaviours, (list-wise deletion) (*n* = 179, 2.36%) as they were perceived to be missing completely at random.

### Participant characteristics

7402 adolescents aged 13–15 years (mean 13.8 ± 0.45 years) was 50.3% female and 71.3% white. Only a small percentage of adolescents were classified as being underweight (1.8%), and therefore, underweight was combined with the normal weight category, due to its low prevalence (Fitzsimons and Pongiglione, 2017).

### Participants dietary typologies

**Figure 1.**
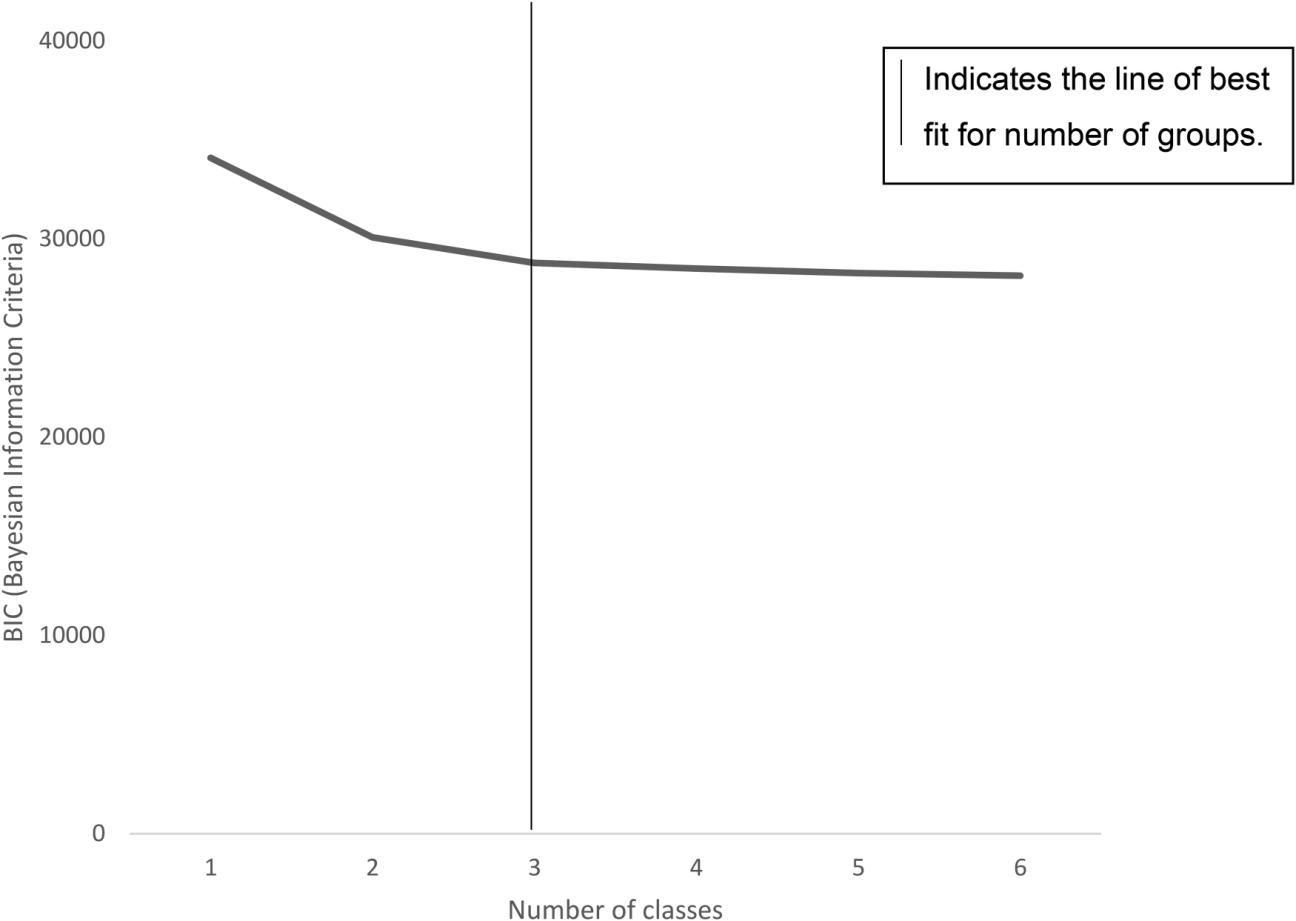
Bayesian information criterion by number of classes.

The class membership of adolescents was inferred from eight dietary behaviours. The model fit criteria were based on the raw BIC score for latent class solutions, with a three-class solution deemed most appropriate, as any solution above this resulted in smaller gains in the model fit. The probabilities for the three classes were 0.83, 0.85 and 0.82 for classes 1 to 3, providing evidence of homogeneity for each subgroup (Figure 1). This means that English adolescents participating in the MCS can be divided into three mutually exclusive groups based on self-reported data on their dietary behaviours.

Three dietary behaviour typologies were identified by the authors (Table 1) while the names are subjective, it was felt these names represent the dietary behaviours within the groups. Class 1 (*n* = 1911 25.82%) was labelled as ‘less-healthy’ with adolescents least likely to consume two portions of fruit, vegetables or breakfast daily but most likely to consume mainly white bread and drink whole milk, SSB and ASB at least daily and FF weekly. There was a higher percentage of boys compared to girls (+2.2%), non-white compared to white ethnicity (+6.0%) and adolescents living with obesity compared to overweight (+3.1%) and healthy weight (+3.1%) in this group. Class 2 (*n* = 2378 32.13%) was defined as ‘healthy’ as adolescents within this group were likely to consume two portions of fruit, two portions of vegetables, or breakfast daily, and least likely to consume SSB and ASB daily, or FF weekly. A higher percentage of girls compared to boys (+5.2%) white compared to non-white ethnicity (+12.7%) and adolescents living with overweight compared to obesity (+5.5%) and healthy weight (+1.6%) were in this group. Class 3 (*n = *3113 42.05%) was labelled as ‘mixed’ as adolescents within this group reported sometimes healthier dietary behaviours, i.e., two portions of fruit and vegetables daily, only sometimes eating breakfast daily, drinking SSB and ASB weekly and eating FF at least once a month, mainly drinking semi-skimmed milk and eating a variety of different types of bread. The demographics of the adolescents within this group were a higher percentage of boys compared to girls (+3.1%), non-white compared to white ethnicity (+6.7%), and adolescents living with obesity compared to overweight (+1.7%) and healthy weight (+0.8%).

**Table 1.** Personal characteristics of each dietary behaviour typology.

		Class 1 (%)	Class 2 (%)	Class 3 (%)
		Less-healthy	Healthy	Mixed
	Participants (%)	*n* = 1911 (25.82)	*n* = 2378 (32.13)	*n* = 3113 (42.05)
Sex	Boy	51.9	45.7	51.6
	Girl	48.1	54.3	48.4
Ethnicity	White	66.9	79.7	68.5
	Non-white	33.1	20.3	31.5
BMI Classification	Obese	21.4	17.4	19.8
	Overweight	13.6	15.2	14.0
	Healthy and under weight	65.0	67.4	66.2
				
				
Fruit (≥ 2 portions a day)	Never	23.1	1	4.1
	Some days	64.7	30.3	81.4
	Everyday	12.1	68.7	14.5
Vegetable (≥ 2 portions a day)	Never	19.9	0	3.9
	Some days	63.6	16.8	77.2
	Everyday	16.6	83.2	19
Breakfast	Never	16.6	2.6	5.7
	Some days but not all days	47	21.7	43.9
	Every day	36.4	75.7	50.4
Sugar-sweetened beverages	Hardly ever or never	12.5	23.2	0.1
	Less than once a month	2.4	13	4
	Less often but at least once a month	3.9	24.9	13.1
	1–2 days a week	5.8	21.4	39.5
	3–6 days a week	11	8.8	34.4
	Once a day	29.7	6.1	7.7
	More than once a day	34.7	2.6	1.2
Artificial-sweetened beverages	Hardly ever or never	29.5	28.1	8
	Less than once a month	4.8	10.6	8.8
	Less often but at least once a month	5.7	20.1	19.7
	1–2 days a week	4.3	18.9	31.3
	3–6 days a week	8.9	7.8	27.3
	Once a day	22.3	7.1	3.9
	More than once a day	24.5	7.4	1
Fast-food	Never	3.5	10.8	0.1
	Less than once a month	10.8	44.6	13.2
	Less often but at least once a month	33.8	40.2	52.9
	1–2 days a week	32.3	4.2	30.6
	3–6 days a week	13	0	3
	Once a day	3.9	0	0.2
	More than once a day	2.7	0	0
Bread	I only eat white bread	53.7	8.4	25.2
	I sometimes eat white bread, sometimes I eat brown or granary or wholemeal bread (including 50:50 bread)	32.8	56.6	61.2
	I only eat brown/granary bread (including 50:50 bread)	6.5	10.3	5.9
	I sometimes eat brown/granary bread (including 50:50 bread), sometimes I eat wholemeal bread	3.7	13.5	5
	I only eat wholemeal bread	2.3	9.9	1.9
	I never eat bread	1.1	1.3	0.7
Milk	I only have whole milk	37.8	10.6	21.1
	I sometimes have whole milk, sometimes I have semi-skimmed or skimmed milk	13.7	14.5	24
	I only have semi-skimmed milk	31.1	49.3	42.7
	I sometimes have semi-skimmed, sometimes I have skimmed milk	2.6	7.9	5.2
	I only have skimmed milk	3.7	5.5	3.2
	I only have 1% fat milk	1.3	2.4	1.2
	I have soya milk or other non-cow milk	0.6	4.9	0.2
	I never have milk	9.3	4.9	2.5

### Socio-ecological determinants of dietary behaviour typologies

Descriptives of the determinants are presented in Table 2. The results of the modelled relationship and the three dietary typologies are presented in Table 3. Mixed typology was chosen as the reference category, as when implementing strategies to improve behaviour it would be useful to think about how adolescents could move from either less healthy to mixed or mixed to healthy typology. Subsequently, all variables were entered into two regression models using a stepwise approach to predict dietary behaviour typologies (Table 4). Model 1, adolescents with an increased likelihood of being within the less-healthy typology versus mixed typology were; white compared to non-white (OR: 0.726, 95% CI = 0.532, 0.992), had a higher intention to attend university (OR: 0.992, 95% CI = 0.987, 0.996), had siblings compared to not having siblings (OR: 1.938, 95% CI: 1.358, 2.766). For each one-unit increase score, the odds of being in the less healthy typology, compared to the mixed typology, decrease for cognitive ability (OR: 0.946, 95% CI = 0.899, 0.996) and increase for social media usage (OR: 1.072, 95% CI = 1.004, 1.141).

**Table 2.** Description of personal characteristics, individual, influential other, social and physical environment variables for adolescents (MCS6)

		Total		Boys		Girls	
		*N*	%	*n*	%	*n*	%
**Personal characteristics**							
Sex		7402	100		49.7		50.3
							
							
Ethnicity	White	5273	71.3	2629	71.4	2644	71.1
	Non-white	2080	28.1	1020	27.7	1060	28.5
BMI	Obese	1377	19.4	700	19.5	677	19.3
	Over-weight	1012	14.3	465	13.0	547	15.6
	Healthy weight	4698	64.4	2420	65.1	2278	59.9
**Individual**			Mean ± SD		Mean ± SD		Mean ± SD
Post-school intention	0–100	7336	87.9 ± 19.3	3641	85.4 ± 21.0	3695	90.4 ± 19.3
University intention	0–100	7107	70.2 ± 28.2	3497	66.4 ± 28.9	3610	73.8 ± 27.0
Cognitive score	0–20	6984	7.13 ± 2.6	3466	7.13 ± 2.7	3518	7.13 ± 2.5
			**%**		**%**		**%**
Perceived weight	Underweight	547	7.5	356	9.9	191	5.2
	About the right weight	4301	59.1	2295	63.8	2006	54.4
	Slightly overweight	2073	28.5	827	23.0	1246	33.8
	Very overweight	360	4.9	117	3.3	243	6.6
Screen time usage	Low	299	4.2	79	2.3	220	6.0
	Medium	3895	55.1	1451	42.4	2444	67.0
	High	2880	40.7	1896	55.3	26.5	27.0
Owning a computer	Yes	6184	83.6	3036	82.5	3148	84.6
	No	1217	16.4	646	17.5	571	15.4
Days spent being physically active/a week	Every day	1314	17.8	890	24.2	424	11.4
	5–6 times	1411	19.1	813	22.1	598	16.1
	3–4 times	2506	33.9	1151	31.3	1355	36.5
	1–2 days	1843	24.9	681	18.5	1162	31.3
	Not at all	322	4.4	146	4.0	176	4.7
Sleep/a night	>10 h	228	3.3	126	3.7	102	2.9
	8–10 h	4727	68.0	2374	68.9	2350	67.1
	<10 h	1994	28.7	945	27.4	1049	30.0
Smoking	Never smoked	6221	85.6	3111	86.6	3110	84.5
	Has smoked	1049	14.4	480	13.4	569	15.5
Well-being	High	3537	48.8	2037	56.9	1500	40.9
	Low	3711	51.2	1540	43.1	2171	59.1
Perceived health	Excellent	920	12.4	609	16.5	311	8.4
	Very good	2727	36.9	1399	38.0	1328	35.7
	Good	2740	37.0	1257	34.2	1483	39.9
	Fair	860	11.6	500	13.5	500	13.5
	Poor	150	2.0	95	2.6	95	2.6
Self-esteem	High	5344	74.2	3021	84.7	2323	63.8
	Low	1863	25.8	544	15.3	1319	36.2
**Influential others**							
Parental status	1 parent/carer	1830	24.7	903	24.5	927	24.9
	≥2 parents/carers	5572	75.3	2779	75.5	2793	75.1
Discipline	High	3733	53.2	1900	55.2	1833	51.3
	Low	3282	46.8	1545	44.8	1737	48.7
Siblings	0	1017	13.7	475	12.9	542	14.6
	≥	6385	86.3	3207	87.1	3178	85.4
							
							
Parent perceived health	Excellent	1447	20.8	751	21.7	696	19.9
	Very good	2314	33.3	1133	32.8	1181	33.8
	Good	2092	30.1	1033	29.9	1059	30.4
	Fair	794	11.4	393	11.4	401	11.5
	Poor	299	4.3	147	4.3	152	4.4
Eating as a family/week	Every day	3003	45.6	1480	45.3	1523	46.0
	Most days	1975	30.0	962	29.4	1013	30.6
	At least once	1403	21.3	722	22.1	681	20.6
	Not at all	199	2.7	105	3.2	94	2.8
Spending time with friends out of school	Most days	2504	33.8	1301	37.0	1203	33.1
	At least once a week	2447	33.1	1214	34.6	1233	34.0
	At least once a month	1350	18.2	593	16.9	757	20.8
	Less than once a month	559	7.6	272	7.7	287	7.9
	Never	284	3.8	133	3.8	151	4.2
Social media/week	None	630	8.5	424	11.5	206	5.5
	<half an hour	956	12.9	639	17.4	317	8.5
	Half-1 h	1089	14.7	651	17.7	438	11.8
	1–2 h	1246	16.8	653	17.7	593	15.9
	2–3 h	1118	15.1	503	13.7	615	16.5
	3–5 h	999	13.5	369	10.0	630	16.9
	5–7 h	712	9.6	235	6.4	477	12.8
	7+ hours	648	8.8	206	5.6	442	11.9
**Social environment**							
Parent education	Higher degree and postgraduate qualifications	535	9.3	260	9.1	275	9.5
	First degree (including B.Ed.)	1197	20.8	593	20.7	604	20.9
	Diplomas in higher education and teaching qualifications	944	16.4	476	16.6	468	16.2
	A/AS levels	500	8.7	241	8.4	259	9.0
	O level/GCSE A-C	1951	33.8	974	33.9	977	33.8
	O level/GCSE < C	637	11.1	327	11.4	310	10.7
Deprivation of area of residence (decile)	1 (most deprived)	764	10.3	396	10.8	368	9.9
	2	758	10.3	389	10.6	369	9.9
	3	632	8.6	322	8.8	310	8.3
	4	955	12.9	482	13.1	473	12.7
	5	591	8.0	280	7.6	311	8.4
	6	764	10.3	375	10.2	389	10.5
	7	733	9.9	364	9.9	369	9.9
	8	775	10.5	377	10.3	398	10.7
	9	693	9.4	324	8.8	369	9.9
	10	726	9.8	366	10.0	360	9.7
			**Mean ± SD**		**Mean ± SD**		**Mean ± SD**
Parent cognition	0–20	6254	10.91 ± 4.5	3112	10.93 ± 4.5	3142	10.89 ± 4.6
			**Median**		**Median**		**Median**
Household income		4476	£33,000–£37,500^^	2239	£37,500–£43,000^^	2237	£33,000–£37,500^^
**Perceived physical environment**							
			**%**		**%**		**%**
Adolescent perception of area safety	Very safe	2176	29.4	1190	32.3	986	26.5
	Safe	4651	62.9	2222	60.4	2429	65.3
	Not very safe	540	7.3	254	6.9	286	7.7
	Not at all safe	30	0.4	13	0.4	17	0.5
Parent perception of area safety	Very safe	2780	42.3	1463	44.8	1317	39.8
	Safe	3306	50.3	1571	48.1	1735	52.5
	Not very safe	433	6.6	206	6.3	227	6.9
	Not at all safe	53	0.8	25	0.8	28	0.8

**Table 3.** Socio-ecological determinants of adolescent dietary behaviour typologies

			Dietary typology – reference mixed typology							
			Less healthy				Healthy			
Independent variable	Responses	Reference category	OR	95% CI		*p* value	OR	95% CI		*p* value
**Personal characteristics**										
Gender	Male	Female	0.974	0.831	1.142	0.746	.923	0.800	1.066	0.276
Ethnicity	White	Non-white	0.773*	0.660	0.906	0.001	2.247**	1.924	2.625	<0.001
BMI	Obese	Healthy and underweight	0.898	0.696	1.160	0.412	0.967	0.766	1.220	0.775
	Obese and Overweight	Healthy and underweight	0.916	0.735	1.142	0.436	1.161	0.955	1.412	0.134
**Individual**										
Post-school intention		Ordinal	0.996*	0.992	1.000	0.047	1.008*	1.003	1.013	0.001
University intention		Ordinal	0.992**	0.989	0.995	<0.001	1.008**	1.005	1.011	<0.001
Perceived weight	Underweight	Very overweight	0.839	0.540	1.305	0.437	0.557*	0.351	0.883	0.013
	About the right weight	Very overweight	0.772	0.531	1.123	0.176	0.804	0.545	1.186	0.271
	Slightly overweight	Very overweight	0.884	0.626	1.247	0.481	0.895	0.622	1.289	0.552
Screen time	Low usage	High usage	1.238	0.800	1.918	0.338	3.820**	2.687	5.430	<0.001
	Medium usage	High usage	0.926	0.798	1.075	0.314	1.753**	1.522	2.081	<0.001
Own a computer		Not owning a computer	1.089	0.898	1.321	0.387	0.850	0.714	1.012	0.067
Physical activity	Every day	Not being physically active	0.597*	0.406	0.877	0.009	1.258	0.805	1.967	0.314
	5–6 days	Not being physically active	0.413**	0.282	0.606	<0.001	1.135	0.731	1.763	0.572
	3–4 days	Not being physically active	0.450**	0.312	0.648	<0.001	1.174	0.764	1.804	0.465
	1–2 days	Not being physically active	0.597*	0.413	0.864	0.006	1.001	0.647	1.551	0.995
Sleep	> 10 h sleep	< 8 h	0.864	0.561	1.332	0.508	1.737*	1.179	2.558	0.005
	8–10 h sleep	< 8 h	0.751**	0.643	0.877	<0.001	1.194*	1.029	1.386	0.019
Not-smoking		Smoking	0.700**	0.578	0.847	<0.001	1.466*	1.18	1.823	0.001
Well-being		Low	0.940	0.801	1.104	0.450	1.242*	1.071	1.439	0.004
Perceived health	Excellent	Poor	0.803	0.479	1.346	0.405	5.475**	2.673	11.217	<0.001
	Very good	Poor	0.650	0.405	1.041	0.073	3.034*	1.512	6.09	0.002
	Good	Poor	0.665	0.420	1.053	0.082	2.048*	1.025	4.092	0.042
	Fair	Poor	0.819	0.509	1.317	0.410	1.638	0.802	3.346	0.176
Self-esteem		Low	0.915	0.762	1.098	0.340	0.783*	0.656	0.934	0.007
Cognitive score		Ordinal data (low to high)	0.927**	0.899	0.956	<0.001	1.131**	1.103	1.161	<0.001
**Influential others**										
Parental status		2 parents	1.376**	1.185	1.599	<0.001	0.731**	0.623	0.857	<0.001
Discipline		Low	0.893	0.781	1.02	0.095	0.875*	0.772	0.992	0.037
Siblings		Not having siblings	1.192	0.976	1.455	0.085	1.279*	1.058	1.545	0.011
Parental perceived health	Excellent	Poor	0.656*	0.477	0.903	0.010	4.006**	2.576	6.231	<0.001
	Very good	Poor	0.793	0.588	1.072	0.131	3.448**	2.233	5.325	<0.001
	Good	Poor	0.749	0.555	1.009	0.058	2.374**	1.533	3.676	<0.001
	Fair	Poor	0.788	0.568	1.095	0.156	1.819*	1.14	2.902	0.012
Family meal frequency	Every day	Not at all	0.755	0.53	1.074	0.118	2.095*	1.306	3.362	0.002
	Most days	Not at all	0.665*	0.464	0.953	0.026	2.107*	1.308	3.394	0.002
	At least once a week	Not at all	0.728	0.505	1.050	0.089	1.592	0.982	2.581	0.059
Spending time with friends	Most days	Not at all	0.754	0.528	1.076	0.120	0.864	0.592	1.259	0.446
	At least once a week	Not at all	0.517**	0.363	0.738	<0.001	1.025	0.707	1.485	0.898
	At least once a month	Not at all	0.501**	0.346	0.724	<0.001	1.127	0.771	1.646	0.537
	Less often than once a month	Not at all	0.699	0.467	1.047	0.082	0.978	0.645	1.481	0.915
Social media		Ordinal data (less to more)	1.089**	1.051	1.128	<0.001	0.819**	0.791	0.848	<0.001
**Social environment**										
Parent education	Higher degree/post grad dip	CSE/GCSE lower than C	0.572*	0.357	0.916	0.020	1.837*	1.208	2.794	0.004
	First degree	CSE/GCSE lower than C	0.536*	0.365	0.789	0.002	2.126**	1.468	3.08	<0.001
	Diploma in HE/teaching qualification	CSE/GCSE lower than C	0.686*	0.485	0.970	0.033	1.417	0.983	2.041	0.061
	A/AS level	CSE/GCSE lower than C	0.672	0.446	1.014	0.058	1.334	0.884	2.013	0.170
	O Level/GCSE A-C	CSE/GCSE lower than C	0.720*	0.536	0.969	0.030	1.130	0.805	1.587	0.481
Parent cognition		Ordinal data (low to high)	0.977	0.950	1.004	0.098	1.078**	1.053	1.104	<0.001
Household income		Ordinal data (low to high)	0.966*	0.942	0.990	0.006	1.053**	1.031	1.077	<0.001
Deprivation	1	10 – least deprived	0.807	0.522	1.248	0.335	0.970	0.696	1.353	0.859
	2	10 – least deprived	1.025	0.671	1.566	0.910	1.014	0.720	1.426	0.938
	3	10 – least deprived	1.438	0.940	2.199	0.094	0.936	0.650	1.348	0.721
	4	10 – least deprived	1.178	0.795	1.746	0.414	0.747	0.535	1.042	0.086
	5	10 – least deprived	1.175	0.745	1.853	0.488	1.161	0.799	1.687	0.434
	6	10 – least deprived	1.209	0.794	1.843	0.377	0.847	0.594	1.210	0.362
	7	10 – least deprived	1.392	0.905	2.142	0.132	1.054	0.732	1.517	0.777
	8	10 – least deprived	1.028	0.674	1.567	0.899	0.806	0.567	1.147	0.231
	9	10 – least deprived	1.233	0.806	1.886	0.335	0.845	0.589	1.212	0.361
	**Perceived physical environment**									
Adolescent perception	Very safe	Not at all safe	0.903	0.339	2.404	0.839	1.137	0.962	1.344	0.133
	Safe	Not at all safe	0.735	0.278	1.941	0.534	0.795	0.294	2.15	0.651
	Not very safe	Not at all safe	1.131	0.423	3.020	0.807	0.470	0.175	1.264	0.135
Parent perception	Very safe	Not at all safe	0.656	0.359	1.200	0.171	0.462	0.169	1.267	0.133
	Safe	Not at all safe	0.817	0.449	1.485	0.507	3.932*	1.470	10.517	0.006
	Not very safe	Not at all safe	0.865	0.463	1.615	0.648	3.170*	1.187	8.462	0.021

OR = odds ratio; *p < 0.05 **p < 0.001.

**Table 4.** Socio-ecological determinants of adolescent dietary behaviour typologies using stepwise approach.

			Dietary typology – reference mixed typology							
			Less-healthy typology				Healthy typology			
Independent variable	Responses	Reference category	OR	95% CI		*p* value	OR	95% CI		*p* value
**Personal characteristics**								
Gender	Male	Female	1.174	0.898	1.535	0.240	0.677**	0.544	0.842	<0.001
Ethnicity	White	Non-white	0.726*	0.532	0.992	0.044	1.339*	1.011	1.774	0.042
BMI	Obese and overweight	Healthy and underweight	0.870	0.677	1.118	0.277	1.553**	1.264	1.915	<0.001
**Individual**										
University intention		Ordinal data	0.992**	0.987	0.996	<0.001	1.005*	1.001	1.009	0.009
Screen time	Low usage	High usage	1.239	0.585	2.623	0.575	2.475*	1.455	4.225	0.001
	Medium usage	High usage	0.892	0.698	1.141	0.364	1.665**	1.348	2.057	<0.001
Physical activity	Every day	Not being physically active	0.652	0.326	1.301	0.225	1.373	0.667	2.825	0.389
	5–6 days	Not being physically active	0.476*	0.249	0.943	0.033	1.229	0.604	2.499	0.570
	3–4 days	Not being physically active	0.536	0.277	1.036	0.064	1.38	0.688	2.768	0.365
	1–2 days	Not being physically active	0.844	0.435	1.638	0.616	1.109	0.546	2.253	0.774
Sleep	> 10 h sleep	< 8 h	1.219	0.542	2.743	0.631	2.731**	1.392	5.356	0.003
	8–10 h sleep	< 8 h	0.813	0.631	1.047	0.109	1.160	0.933	1.442	0.181
Not-smoking		Smoking	0.796	0.572	1.109	0.178	1.332	0.958	1.853	0.088
Wellbeing		Low	0.811	0.634	1.038	0.097	1.147	0.937	1.403	0.183
Perceived health	Excellent	Poor	0.784	0.321	1.914	0.594	4.143*	1.355	12.666	0.013
	Very good	Poor	0.674	0.297	1.529	0.345	2.128	0.718	6.306	0.173
	Good	Poor	0.664	0.296	1.491	0.321	1.451	0.491	4.289	0.501
	Fair	Poor	1.099	0.471	2.567	0.827	1.428	0.463	4.409	0.536
Cognitive ability		Ordinal data	0.946*	0.899	0.996	0.034	1.076**	1.035	1.120	<0.001
**Influential others**									
Siblings		Not having siblings	1.938**	1.358	2.766	<0.001	1.087	0.804	1.468	0.588
Social media		Ordinal data (less to more)	1.072*	1.004	1.141	0.038	0.863**	0.817	0.911	<0.001
**Social environment**										
Higher degree/post grad dip		CSE/GCSE lower than C	0.743	0.416	1.329	0.317	1.686*	1.023	2.785	0.041
First degree		CSE/GCSE lower than C	0.819	0.512	1.309	0.404	1.959*	1.258	3.052	0.003
Diploma in HE/teaching qualification		CSE/GCSE lower than C	0.877	0.588	1.377	0.628	1.568*	1.013	2.426	0.044
A/AS level		CSE/GCSE lower than C	0.979	0.593	1.618	0.935	1.415	0.869	2.303	0.163
O Level/GCSE A-C		CSE/GCSE lower than C	0.904	0.623	1.322	0.574	1.241	0.827	1.864	0.297
Parent cognition		Ordinal data	0.973	0.945	1.008	0.129	1.073**	1.043	1.105	<0.001
Household income		Ordinal data	0.983	0.953	1.014	0.269	1.043*	1.016	1.071	0.002

OR = odds ratio; *p < 0.05 **p < 0.001.

Model 2, adolescents with an increased likelihood of being within the healthy typology versus mixed typology were; male compared to female (OR: 0.677, 95%CI = 0.544, 0.842), being white ethnicity compared to non-white (OR: 1.339, 95% CI = 1.011, 1.774), living with obese and overweight compared to not (OR: 1.553, 95% CI = 1.264, 1.915), both low (OR: 2.475, 95% CI = 1.455, 4.225) and medium (OR: 1.665, 95% CI = 1.348, 2.057) screen usage compared to high usage, having greater than 10 hours sleep compared to <8 hours (OR: 2.731, 95% CI = 1.392, 5.356) and parental education, having a higher degree/post graduate diploma (β = 1.686, 95% CI = 1.023, 2.785) and first degree (β = 1.959, 95% CI = 1.258, 3.052) compared to CSE/GCSE lower than grade C. For each one-unit increase score, the odds of being in the healthy typology, compared to the mixed typology, increased for having higher intention to attend university (OR: 1.005, 95% CI = 1.001, 1.009), higher parent cognition (OR: 1.073, 95% CI = 1.043, 1.105) higher household income (OR: 1.043, 95% CI = 1.043, 1.016), whilst decrease for higher social media usage (OR: 0.863, 95% CI = 0.817, 0.911).

### Path analysis of socio-ecological determinants on dietary behaviour typologies

Variables that were statistically significant (*p* < 0.05) in the stepwise analysis were included in the path analysis. Initial path analyses were performed, and estimates were calculated for different combinations of dietary typology (Table 5). Whilst the magnitude of coefficients is relatively small, statistically significant associations between less-healthy and mixed dietary behaviour typology were university intention (coefficient β = 0.004, 95% CI = 0.002, 0.005), physical activity (β = −0.074, 95% CI = −0.115, −0.033), and having siblings (β = 0.246, 95% CI = 0.105, 0.387). When comparing healthy and mixed dietary behaviour typologies the most significant associations included university intention (β = −0.002, 95% CI = −0.002, −0.001), screen time (β = 0.104, 95% CI = 0.067, 0.141), cognitive ability (β = −0.016, 95% CI = −0.024, −0.008), social media usage (β = 0.035, 95% CI = 0.024, 0.046), parent qualification (β = 0.027, 95% CI = 0.012, 0.041) and parent cognitive ability (β = −0.013, 95% CI = 0.018, −0.007).

**Table 5.** Path analysis of socio-ecological variables on dietary behaviour typologies (reference less healthy).

	Dietary typologies											
	Less healthy and mixed			Healthy and mixed			Less-healthy and healthy			Less-healthy, healthy and mixed		
Independent Variable	Coefficient	95% CI		Coefficient	95% CI		Coefficient	95% CI		Coefficient	95% CI	
Characteristics												
Gender	0.075	−0.025	0.174	−0.064*	−0.107	−0.020	0.062*	0.018	0.106	0.002	−0.060	0.064
Ethnicity	−0.158*	−0.275	−0.041	−0.080*	0.025	0.134	−0.104**	−0.158	−0.050	−0.056	−0.132	0.019
BMI	−0.010	−0.103	0.083	−0.060	0.019	0.101	−0.063*	−0.105	−0.021	−0.013	−0.046	0.071
Individual												
University intention	0.004**	0.002	0.005	−0.002**	−0.002	−0.001	−0.002**	−0.002	−0.004	−0.002*	0.001	0.003
Screen time	−0.037*	0.121	0.046	0.104**	0.066	0.141	−0.076**	−0.114	−0.038	−0.037	−0.016	0.090
Physical activity	−0.074**	−0.115	−0.001	0.029*	0.010	0.047	−0.047**	−0.066	−0.029	−0.020	−0.049	0.003
Sleep	−0.091*	−0.180	−0.001	−0.009*	−0.014	−0.004	−0.067*	−0.106	−0.027	−0.012	−0.068,	0.045
Cognitive ability	0.028*	0.009	0.047	−0.016**	−0.024	−0.008	−0.016**	0.008	0.024	0.004	−0.007	0.015
Influential others												
Siblings	0.246*	0.105	0.387	0.028	−0.033	0.089	0.057	−0.001	0.115	0.124*	0.034,	0.210
Social media	−0.028*	−0.052	−0.004	0.035**	−0.052	−0.004	−0.035**	−0.046	−0.025	0.002	−0.013	0.017
Social environment												
Parent qualification	−0.015	−0.047	0.017	0.027**	0.012	0.041	−0.024*	−0.039	−0.009	0.006	−0.014	0.027
Parent cognitive ability	0.011	−0.002	0.024	−0.013**	−0.018	−0.007	0.014**	0.008	0.020	−0.001	−0.009	0.007
Household income	0.010	−0.001	0.022	−0.009*	−0.014	−0.004	0.012**	0.007	0.017	0.002	−0.006	0.009

Coefficient (95% CI). *p < 0.05 **p < 0.001

All pathways drawn between the less-healthy and healthy typologies demonstrated significant associations except for siblings. Gender (β = 0.062, 95% CI = 0.018, 0.106), ethnicity (β = −0.104, 95% CI = −0.158, −0.050), BMI (β = −0.063, 95%CI = −0.105, −0.022), university intention (β = 0.003, 95% CI = 0.002, 0.003), screen time (β = −0.076, 95% CI = 0.002, 0.004), physical activity (β = −0.047, 95% CI = −0.066, −0.029), sleep (β = 0.016, 95% CI = −0.016, −0.027), cognitive ability (β = 0.016, 95% CI = 0.008, 0.024), social media usage (β = −0.035, 95% CI = −0.046, −0.025), parent qualifications (β = −0.024, 95% CI = −0.034, −0.009), parent cognition (β = 0.014, 95% CI = 0.008, 0.020) and income (β = 0.012, 95% CI = 0.007, 0.017) were all related to the extremes for dietary typologies.

The final path analysis (Table 5) used all three dietary behaviour typologies as dependent variables. Only two determinants reached statistical significance: university intention (β = 0.002, 95% CI = 0.001, 0.003) and siblings (β = 0.124, 95% CI 0.048, 0.209). Further model development including gender, ethnicity, and BMI as possibilities for predicting the variance of other determinants in the model was attempted, however, the model fit was poor.

## Discussion

This study used the concept of a systems framework to consider the clustering of dietary behaviours and socio-ecological determinants in a national sample of adolescents. Path analysis was conducted to assess the estimates of the magnitude and significance of hypothesised connections between socio-ecological predictors and dietary typologies. Analyses revealed three typologies of dietary behaviours: healthy, less-healthy and mixed, with clear distinctions of dietary behaviours between them. The mixed dietary typology had the largest percentage (42.05%) of adolescents, which acknowledges many participants identified with a combination of dietary behaviours (e.g., consuming fruit, vegetables, but also FF and SSB). It is important to recognise when interpreting the magnitude of coefficients within the path analysis presented in this study, there is often only a small to moderate effect. A coefficient with a magnitude around 0.1 or less may suggest a relatively weak relationship between the variables. By examining associations with different combinations of dietary behaviour typology, it was perhaps not surprising, that depending on the typologies entered into the model, the coefficients altered. Small effects can be statistically significant, especially in large sample sizes, and may be meaningful in the context of recognising the importance of targeting multiple behaviours if we are to improve adolescent dietary behaviours.

To target health behaviour change and recognise the importance of a systems approach (Bagnall et al., 2019; Rutter et al., 2017), it is essential to consider multiple socio-ecological layers simultaneously. Indeed, the findings presented in this study illustrate that influences on adolescent dietary behaviour are derived from multiple socio-ecological layers. Personal characteristics, such as gender, ethnicity, and BMI, had one of the smallest impacts on typologies compared to the other layers. This is important in recognising that the influence of dietary behaviours goes beyond the individual. Indeed, these findings highlight the role that society may have in positive adolescent development including restricting screen time, encouraging physical activity, developing cognitive ability, and promoting a positive social environment.

Targeting multiple socio-ecological layers simultaneously recognises external influences on adolescent behaviours. This study supports the findings of previous studies reporting an association between family meal frequency and healthier dietary behaviours (Levin et al., 2012; Pearson et al., 2009; Stewart and Menning, 2009). However, as part of the family influence, this study included having siblings as a potential influence and found that this was positively associated with being in the mixed dietary behaviour typology, when compared to the less-healthy typology, although statistical significance did not extend to the healthy typology. The role of siblings in dietary behaviours has not been widely reported, possibly due to the challenge, if not impossible, of changing this determinant. However, siblings play a part in the family structure and therefore, future studies should investigate the complex relationship and mechanisms between parental marital status, and family structure, including siblings. Current interventions are often aimed at families and focus on consuming meals as a family (Dwyer et al., 2015), however, family-based interventions to address the intake of less-healthy foods may be useful for improving adolescent dietary behaviours (Bogl et al., 2017).

A modern external influence on adolescent dietary behaviours is the use of social media. Our findings corroborate those of other studies (Finger et al., 2015; Kenney and Gortmaker, 2017; Lipsky and Iannotti, 2012), about the increased use of screen time having a negative impact on dietary behaviours. Social networking sites have successfully been used as a tool for delivering healthcare education and interventions (Laranjo et al., 2014). Given their popularity among teens, promoting healthy eating via these sites may provide a platform to positively change behaviours. Future research should clarify the independent contributions of different types of screen usage on dietary behaviours.

This study found an association between parental education, household income, and food choice. Notably, adolescents with parents with higher cognitive scores and higher household income were more likely to have healthier dietary behaviours. Despite Darmon and Drewnowski (2015) suggesting that socioeconomic disparities in diet quality may be explained by the higher cost of healthy diets and the potential inaccessibility of fresh whole foods in some neighbourhoods, Laraia et al. (2017) suggest that the relationship is more complex. They suggest that the burdens of uncertainty with employment, food and housing, in families with lower incomes, can threaten well-being leading to psychological and cognitive burden. These in turn can influence bio-behavioural pathways (e.g., psychological distress, short sleep duration), subsequently predisposing to poorer dietary behaviours.

In contrast to previous literature (Moore and Littlecott, 2015; Niven et al., 2013) and other social environment factors, deprivation measured by deciles of IMD, was not associated with dietary behaviours. These findings may be a result of considering deprivation at an area level (i.e., postcode), which suggests that adolescents within deciles of IMD have the same characteristics and is not sensitive enough as a measure. Further sensitive measures of deprivation, for example, the MacArthur ladder SES and perceived SES, beyond deciles may be more informative, as per findings from other literature (Tan et al., 2020). However, the data does imply that there is an urgent need for effective social and public health policies to tackle socioeconomic inequalities in dietary behaviours.

The strengths of this study include the consideration of a wide range of socio-ecological determinants (personal characteristics, individual, influential others, social and perceived physical environment), the large national sample size, and the ability to determine the relative influence of factors at multiple levels individually and in combination. However, limitations include the use of self-reported cross-sectional data, presented in this study which was restricted to a narrow age range (14 years) and ethnic grouping, therefore inference of the results to other age groups or smaller ethnic minorities may be problematic. However, mid-adolescence is an important age to potentially influence behaviours therefore, it is important to assess dietary behaviours given the links to future health (Afshin et al., 2019). The limited number of dietary behaviour questions, with no indication of portion sizes, and the potential influence of social desirability bias could lead to a difference in the interpretation of the questions. However, the large sample size, representing all SES groups provided a good reflection of the general population of English adolescents, allowing for typologies of dietary behaviour to be established.

The current analysis was based on a rich source of national data, however, the contribution of socio-ecological variables that could be included within the model for each layer depended on those available as part of the Millennium Cohort Study. For example, it may have been that the physical environment as a socio-ecological layer has a higher level of importance but was limited by the measures that were available. The lack of data on the perceived availability of food to the participants needs to be acknowledged as previous literature has indicated this is an important determinant of eating behaviours (Pearson et al., 2009). The data was collected in 2014 and subsequently published by the centre for longitudinal studies in 2017, due to the time required to process the data. Since 2014, there has been an increase in social media platforms as well as screen time opportunities. As such, these changes may have a substantive impact on adolescent dietary behaviours and therefore require further investigation in future studies.

## Conclusion

This study reported how three distinct dietary behaviour typologies were associated with multiple socio-ecological factors, recognising the importance of a systems approach for improving adolescent dietary behaviours. Notable findings include: (i) the inverse relationship between screen time, social media usage, and healthy dietary behaviours; (ii) the inverse relationship between intention to attend University and having siblings and less-healthy dietary behaviours and (iii) a positive relationship between cognition, parent cognition and household income and the healthy dietary behaviours. These findings were supported by path analysis, which identified that intention to attend university, screen time and social media usage, physical activity, adolescent and parent cognition, and parental qualification were most significantly associated with dietary behaviour typology. It should be noted that we are at risk of over-interpretation of findings if we do not recognise that these findings altered depending on the combination of typologies entered into the model; however, they highlight the need to move away from silo behaviours on individual dietary behaviours, and a step towards more systems thinking to tackle dietary behaviour change.
